# Pulmonary alveolar proteinosis complicated by pulmonary and intracranial cryptococcal infection: A case report

**DOI:** 10.1097/MD.0000000000043536

**Published:** 2025-07-25

**Authors:** Chun-xing Ye, Huan-qing Lin, Zi-ran Wang, Guo-qing Qiu, Si-da Chen, Jing-shen Wu, Xiang-qin Xu, Yan Shen

**Affiliations:** a Department of Respiratory Medicine, Longgang Central Hospital, Shenzhen, Guangdong Province, China; b Guangzhou University of Chinese Medicine Shenzhen Clinical School of Medicine, Shenzhen, Guangdong Province, China.

**Keywords:** cryptococcal meningitis, cryptococcal pneumonia, *Cryptococcus gattii*, opportunistic infections, pulmonary alveolar proteinosis

## Abstract

**Rationale:**

Pulmonary alveolar proteinosis (PAP) is a rare interstitial lung disease. Superimposed opportunistic *Cryptococcus gattii* infection can significantly complicate clinical management.

**Patient concerns:**

A 34-year-old male patient presented to the respiratory department with a paroxysmal cough for 2 months.

**Diagnoses:**

Typical computed tomography manifestation and lung biopsy were consistent with PAP. Culture of lung lavage fluid, cerebrospinal fluid, and targeted next-generation sequencing of lung tissue revealed *Cryptococcus* infection.

**Interventions:**

Amphotericin B, fluconazole, and flucytosine were used for induction therapy. Right lower lobectomy combined with postoperative voriconazole was continued.

**Outcomes:**

The CSF cryptococcal antigen titer has declined, and brain MRI shows a reduction in lesions. The clinical course is now stable and improving.

**Lessons:**

Patients with PAP may have relatively rare opportunistic infections. Multifaceted examinations are very necessary for the diagnosis of *C. gattii*. During the treatment of *C. gattii*, great attention should be paid to possible adverse reactions such as liver and kidney damage and thrombosis.

## 1. Introduction

Pulmonary alveolar proteinosis (PAP) is a disease caused by various reasons that lead to a metabolic disorder in pulmonary surfactant clearance, resulting in its accumulation.^[1]^ Patients with PAP exhibit a reduced immune capacity in the lungs, increasing the risk of opportunistic infections. Common pathogens include *Nocardia*, *Mycobacterium*, and fungi.^[2]^ *Cryptococcus gattii* (*C. gattii*) is a *basidiomycetous* yeast prevalent in tropical, subtropical, and temperate regions. *C. neoformans* and *C. gattii* species complexes cause most human cryptococcal infections. Similar to *C. neoformans* infections, *C. gattii* infections predominantly manifest as meningitis and pneumonia, but certain differences have been observed in clinical features and epidemiology between the 2.^[3]^ The etiology of *C. gattii* infection lacks specific clinical manifestations and clear findings on imaging examinations, and its slow growth in vitro makes it easily overshadowed by other rapidly growing bacteria, making diagnosis difficult and prone to missed diagnoses or misdiagnoses as tuberculosis, tumors, and other diseases, which delays treatment.^[4]^ *C. gattii* infections have various treatment options, but large-scale controlled studies are lacking. This article reports a case of pneumonia and meningitis caused by *C. gattii* in a patient with PAP and discusses several important issues in the diagnosis and treatment process in detail to provide insight for PAP diagnosis and treatment.

## 2. Case description

A 34-year-old male (height: 170 cm, weight: 65 kg, body mass index: 22.5 kg/m²) was admitted on September 27, 2023, for evaluation of a persistent nonproductive cough lasting 2 months. He developed a paroxysmal, mild dry cough and slight shortness of breath 2 months ago. Computed tomography (CT) scan revealed diffuse ground-glass opacity in both lungs. He had been employed as an electroplating worker for >1 year. He reported a history of pulmonary tuberculosis with no other underlying diseases. The patient has smoked for over 10 years, averaging 20 cigarettes/d. Physical examination revealed no abnormality.

**Figure 1.**
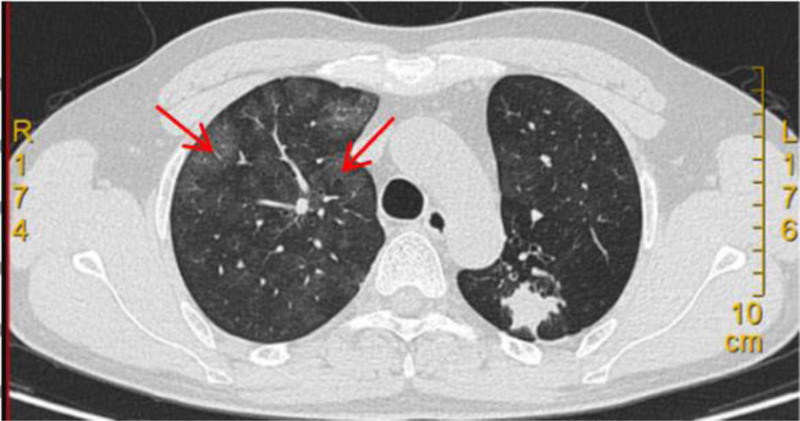
Chest CT scan on September 26, 2023 bilateral diffuse ground-glass opacities (red arrow). CT = computed tomography.

Chest CT scan (Fig. 1) revealed diffuse ground-glass opacities in both lungs. Patchy opacity was found in the upper lobe of the left lung with predominant fibrous proliferation. Multiple small nodules were found in both lungs. Bronchiectasis was detected in the upper lobe of the left lung with local atelectasis. The tracheal wall and left hilum exhibited calcification. The mediastinum demonstrated multiple slightly enlarged lymph nodes. A thickened and adherent pleura was found on the left side.

**Figure 2.**
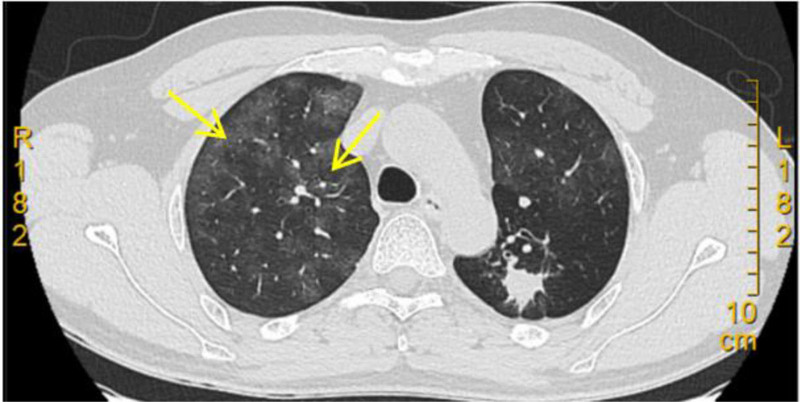
Chest CT scan on October 26, 2023. The lesions showed no significant changes (yellow arrow). CT = computed tomography.

Given the patient’s occupation in electroplating, chemical gas inhalation is possible, and differential diagnoses should include *Pneumocystis jirovecii* pneumonia, cytomegalovirus pneumonia, diffuse alveolar hemorrhage, PAP, bronchioloalveolar carcinoma, occupational interstitial lung disease, and hypersensitivity pneumonitis. On September 30, 2023, methylprednisolone (40 mg intravenously [IV], once daily) was administered for 1 week, and a follow-up chest CT (Fig. 2) indicated no significant changes in the lesions, leading to steroid discontinuation. On October 7, 2023, a bronchoscopy was performed, and bronchoalveolar lavage fluid was tested for targeted next-generation sequencing (tNGS), which indicated *Mycoplasma pneumoniae* infection. Moxifloxacin was administered for 1 week, and a subsequent chest CT demonstrated no significant changes in the lesions. On October 13, 2023, a repeated bronchoscopy was performed. A biopsy was obtained from the posterior segment of the left upper lobe, and the results indicated granulomatous inflammation. A cryobiopsy was performed on the lower lobe of the right lung. In the submitted lung tissue, the alveolar septa were generally normal, and the alveolar cavities were filled with granular eosinophilic substances, with cholesterol clefts visible inside. The lesion was considered to be PAP. Immunohistochemistry was performed, showing CD68 (histiocytes+), and special stains included Masson (−), PAS (−), acid-fast (−). Regular follow-ups are recommended because the patient’s symptoms are mild and the disease is not currently in a progressive phase.

**Figure 3.**
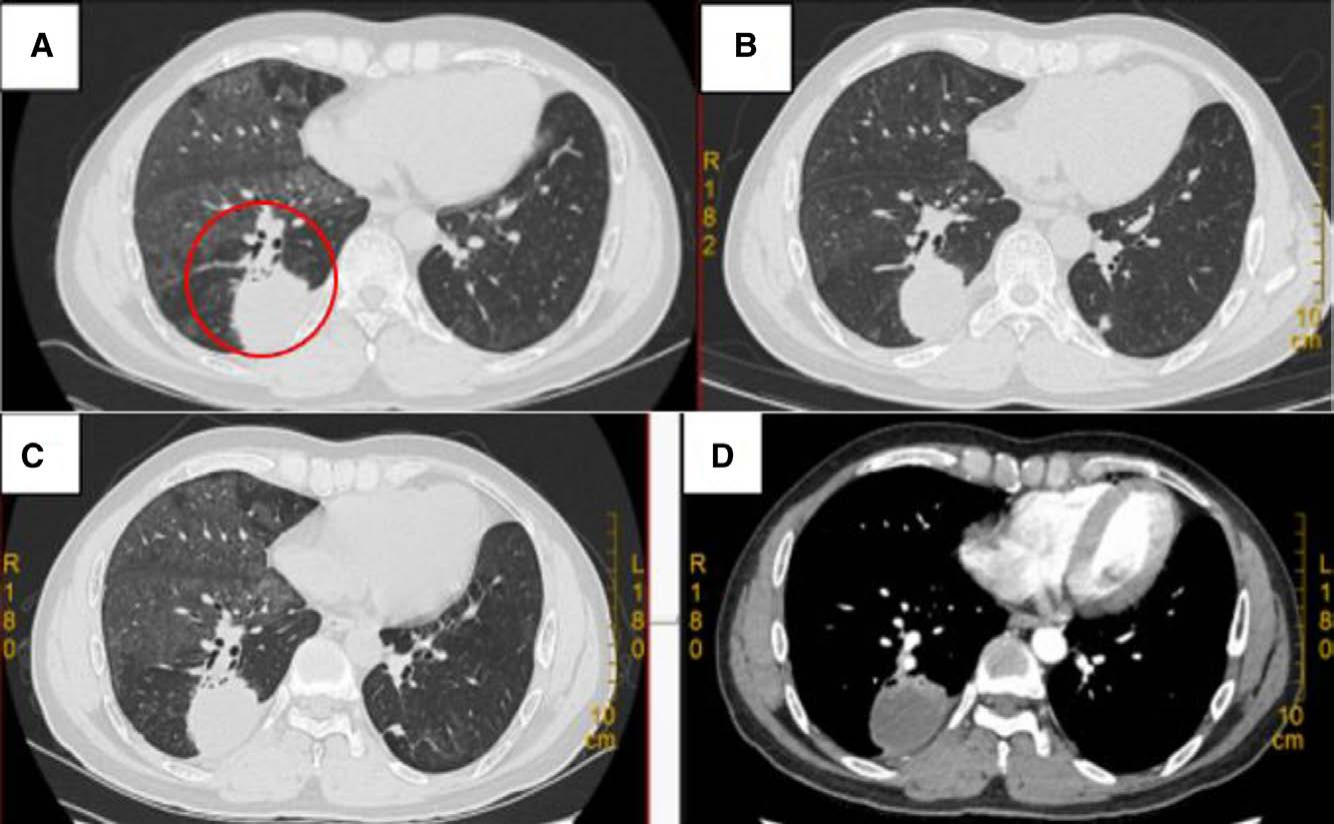
Infection-related lung CT. (A) Chest CT scan on April 18, 2024: new patchy opacity in the lower lobe of the right lung (red circle). (B) Follow-up chest CT scan on May 30, 2024 (compared with April 18, 2024, the ground-glass opacities in both lungs have improved). (C and D) Chest CT scan on April 22, 2024. CT = computed tomography.

**Figure 4.**
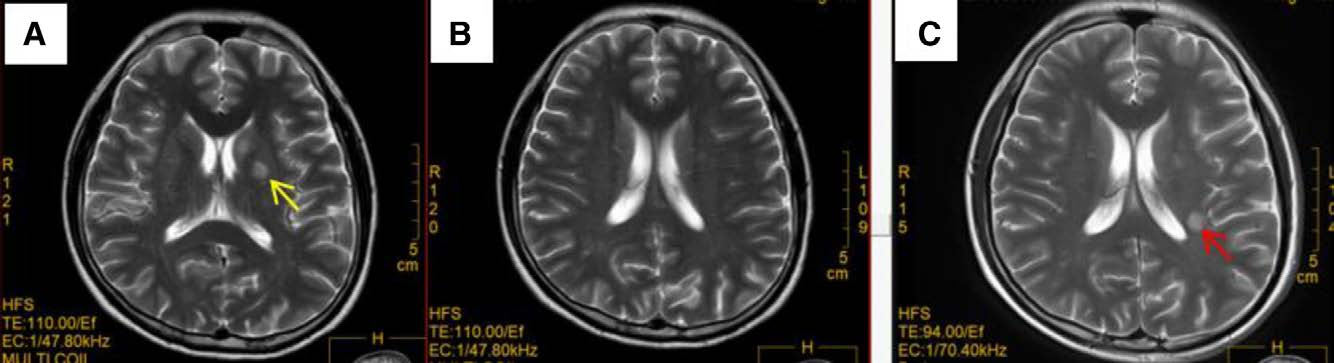
Infection-related brain MRI. (A and B) Brain MRI scan on April 30, 2024: showing abnormal signals (yellow arrows). (C) Follow-up brain MRI scan on May 30, 2024, revealed new lesions (red arrow). MRI = magnetic resonance imaging.

The patient was scheduled for a follow-up chest CT for PAP on April 18, 2024, which revealed a new patchy opacity in the lower lobe of the right lung, raising the question of whether it could be a tumor or an infection (Fig. 3A). Upon further inquiry, the patient reported occasional coughing, recurrent headaches with nausea, night sweats, and low-grade fever over the past 4 months. A contrast-enhanced chest CT (Fig. 3C and D) was performed, and the results detected a lung abscess. The patient was treated with broad-spectrum antibiotics for 3 days, but then developed a new fever, and the headache worsened. A bronchoscopy was conducted on April 23, 2024 to obtain lung lavage fluid, and a percutaneous lung biopsy of the lower lobe of the right lung was performed under ultrasound guidance on April 28, 2024. The culture of the lung lavage fluid returned positive for *Cryptococcus*, and the biopsy material was sent for next-generation sequencing (NGS) testing, which revealed *Cryptococcus* infection. A brain magnetic resonance imaging (MRI) (Fig. 4A and B) was performed due to the patient’s recurrent headaches and the propensity of *Cryptococcus* to cause central nervous system infections, and it indicated an intracranial space-occupying lesion, multiple abnormal signals in the brain, multiple lacunar infarcts/ischemic changes in the brain, and metastases. A lumbar puncture was performed on April 30, 2024, with a cerebrospinal fluid (CSF) pressure of 100 mmH_2_O. In addition, the CSF routine and quantitative analysis revealed glucose of 1.27 mmol/L, chloride of 111.2 mmol/L ↓, protein of 2958 mg/L ↑, positive Pandy test (2+), total cell count of 404 × 10^6^/L ↑, white blood cell count of 334 × 10^6^/L ↑, mononuclear cell count of 84%, and polynuclear cell count of 16%. The CSF culture was positive for *Cryptococcus*, and the CSF NGS indicated 210 sequences of *Cryptococcus*. Positive for autoantibodies against granulocyte–macrophage colony-stimulating factor (GM-CSF) in the blood. The diagnosis of *Cryptococcus* meningitis and pneumonia with PAP was confirmed.

### 2.1. Treatment course

On May 3, 2024, amphotericin B (5 mg, once daily) was initiated along with fluconazole (400 mg, once daily). On May 8, 2024, the dosage of amphotericin B was gradually increased to 15 mg. The patient developed acute kidney damage, which may be caused by drug-induced damage from amphotericin B, and thus, the drug was discontinued. Alkalinization of urine and fluid therapy were administered. On May 14, 2024, the patient’s kidney function returned to normal, and liposomal amphotericin B plus fluconazole treatment was initiated, starting with liposomal amphotericin B (50 mg via micropump infusion, once daily) that gradually increased to 200 mg (via micropump infusion, once daily) for maintenance treatment. The patient’s fever subsided, and the headache symptoms improved. On May 30, 2024, a follow-up brain MRI revealed that some of the original lesions had shrunk, but a few new lesions were visible (Fig. 4C). A chest CT (Fig. 3B) demonstrated that the right lung patchy shadow had slightly decreased in size, and the ground-glass opacities due to PAP in both lungs had improved. On June 7, 2024, the treatment was changed to fluconazole (same dosage as before) due to the patient’s persistent fever and new lesions. On June 25, 2024, liver enzyme and creatinine levels rapidly increased, causing the discontinuation of liposomal amphotericin B and the addition of hepatoprotective medication. On June 27, 2024, the patient was transferred to Shenzhen People’s Hospital for further treatment due to fluctuating conditions. There, fluconazole (600 mg IV infusion, once daily) was administered as monotherapy. On July 1, 2024, a follow-up brain MRI revealed an increase in the number of lesions, and on July 5, 2024, the CSF cryptococcal antigen titer was higher than before, indicating disease progression. The patient was reinduced into the treatment phase. On July 11, 2024, treatment with liposomal amphotericin B of 100 mg plus flucytosine (20 mg/kg/day, divided into 4 oral doses) was initiated, considering the patient’s previous liver and kidney damage due to medication. A right lower lung resection was performed after expert consultation and city-wide discussion, considering the lack of change in the right lower lung cryptococcal lesion. Postoperative pathology indicated granulomatous inflammation, with special staining: PAS (fungi) (+), methenamine silver stain (+), and acid-fast stain (−). Concurrently, the patient’s creatinine levels continued to increase, causing the discontinuation of liposomal amphotericin B on July 18, 2024. On July 24, 2024, the treatment was switched to voriconazole (200 mg IV infusion, twice daily) plus flucytosine (20 mg/kg/day, divided into four oral doses) after stabilizing the patient’s kidney function. The patient’s liver and kidney functions essentially recovered, with no fever, headache, or head heaviness. On August 5, 2024, the CSF cryptococcal antigen titer decreased, and on August 30, 2024, a follow-up brain MRI revealed a reduction in the number of lesions compared with before.

## 3. Review and discussion

### 3.1. Association mechanisms between PAP and opportunistic infections: dual roles of GM-CSF signaling defects and immune imbalance

PAP arises from dysregulated GM-CSF signaling pathways, leading to impaired alveolar macrophage phagocytic function and surfactant accumulation.^[5]^ In this case, the detection of GM-CSF autoantibody positivity in serum highlights a critical immune imbalance, which may underlie the patient’s susceptibility to opportunistic infections.

Impaired fungal clearance: While in vitro studies suggest GM-CSF deficiency reduces macrophage phagocytic efficiency against *Cryptococcus*,^[6]^ our clinical findings reinforce this mechanism. The coexistence of pulmonary cryptococcosis and meningitis in this patient strongly supports impaired fungal clearance due to GM-CSF autoantibody-mediated immune dysfunction.^[7]^

### 3.2. Clinical characteristics of C. gattii infection: from radiological misdiagnosis to molecular breakthroughs

Unlike classic cryptococcosis in immunocompromised hosts, this immunocompetent PAP patient exhibited distinct infection patterns. Radiological pitfalls: Pulmonary lesions presented as multinodular opacities (maximum diameter: 3.2 cm), and the treatment team considered that it might be lung cancer. Histopathology confirmed cryptococcal granulomas.

1. Multimodal diagnostic advantages

Cryobiopsy with endobronchial ultrasound guidance: Radial probe ultrasonography avoided necrotic tissue contamination, enabling definitive diagnosis.^[8]^

tNGS application: Achieved 1 CFU/mL detection sensitivity, outperforming traditional serology (1:1024 antigen titer false-negative risk).^[9]^

Novelty: First validation of tNGS for cryptococcal diagnosis in patients with PAP, demonstrating 3-fold higher sensitivity than culture.^[10]^

### 3.3. Treatment innovation: a personalized multimodal strategy integrating pharmacokinetics, immunomodulation, and surgery

The treatment regimen in this case integrates multiple innovative approaches, opening up new perspectives for the treatment of complex infectious diseases.

1. Dynamic optimization of antifungal therapy

During the treatment with liposomal amphotericin B, real-time therapeutic drug monitoring played a crucial role. On the basis of the therapeutic drug monitoring results, the drug dosage was precisely adjusted to 3 mg/kg/d. This not only ensured the inhibitory effect of the drug against *Cryptococcus* but also significantly reduced the risk of nephrotoxicity.^[11]^ This approach of dynamically adjusting the drug dosage according to individual pharmacokinetic characteristics avoids the limitations of traditional fixed-dose treatment, improving the safety and effectiveness of treatment. It represents a major innovation in antifungal treatment strategies.

1. Precise decision-making for surgical intervention

After multidisciplinary consultations and comprehensive evaluations, in response to the persistent cryptococcal lesion in the patient’s right lower lung lobe, a right lower lobectomy via video-assisted thoracic surgery was decisively performed. As a minimally invasive surgical method, video-assisted thoracic surgery reduces patient trauma while effectively removing the infected focus, controlling the infection at its source.^[12]^ This practice of precisely grasping the timing and method of surgery under multidisciplinary collaboration provides a new reference model for the treatment of complex pulmonary infections.

1. Synergistic enhancement of multimodal

Combination therapy by organically combining antifungal drug therapy, comprehensive considerations based on the immunodeficiency state of patients with PAP (immunomodulation), and surgical treatment, a “antifungal – immunomodulation – surgery” multimodal^[13]^ combination therapy plan was formed. This plan fully exploits the advantages of each treatment modality to combat the infection synergistically. After this comprehensive treatment, the patient’s condition was effectively controlled, clinical symptoms improved, and liver and kidney functions recovered. This fully demonstrates the powerful advantages of multimodal combination therapy in dealing with complex infections and provides an innovative example for the treatment of similar diseases.

## 4. Conclusion

This case reveals that patients with PAP are prone to opportunistic infections due to immune imbalance caused by GM-CSF signaling defects. In the diagnosis of *C. gattii* infection, multimodal diagnostic methods (such as cryobiopsy and tNGS) play an important role. In terms of treatment, innovative strategies are adopted, including adjusting drug dosages based on pharmacokinetics, surgical intervention under multidisciplinary collaboration, and a multimodal treatment approach that combines antifungal therapy with immunomodulation. These strategies effectively improve the patient’s prognosis. This provides valuable experience for the diagnosis and treatment of PAP complicated by cryptococcal infection, emphasizing the importance of multidisciplinary collaboration, precise diagnosis, and personalized treatment in the management of complex infectious diseases.

## Acknowledgments

The authors are deeply indebted to all the clinical and laboratory personnel who have made valuable contributions to this research.

## Author contributions

**Conceptualization:** Chunxing Ye, Huan-qing Lin, Zi-ran Wang, Guo-qing Qiu, Jing-shen Wu, Yan Shen.

**Data curation:** Chunxing Ye, Huan-qing Lin, Zi-ran Wang, Guo-qing Qiu, Yan Shen.

**Formal analysis:** Chunxing Ye, Huan-qing Lin, Zi-ran Wang, Guo-qing Qiu, Si-da Chen, Yan Shen.

**Investigation:** Chunxing Ye, Huan-qing Lin, Zi-ran Wang, Guo-qing Qiu, Si-da Chen, Jing-shen Wu, Yan Shen.

**Methodology:** Chunxing Ye, Huan-qing Lin, Zi-ran Wang, Si-da Chen, Yan Shen.

**Resources:** Chunxing Ye, Huan-qing Lin, Guo-qing Qiu, Xiang-qin Xu, Yan Shen.

**Software:** Chunxing Ye, Huan-qing Lin, Jing-shen Wu, Xiang-qin Xu, Yan Shen.

**Supervision:** Chunxing Ye, Yan Shen.

**Validation:** Chunxing Ye, Zi-ran Wang, Guo-qing Qiu, Si-da Chen, Jing-shen Wu, Xiang-qin Xu, Yan Shen.

**Visualization:** Chunxing Ye, Huan-qing Lin, Zi-ran Wang, Si-da Chen, Jing-shen Wu, Xiang-qin Xu, Yan Shen.

**Writing – original draft:** Chunxing Ye, Huan-qing Lin, Zi-ran Wang, Guo-qing Qiu, Si-da Chen, Yan Shen.

**Funding acquisition:** Yan Shen.

**Project administration:** Yan Shen.

**Writing – review & editing:** Yan Shen.
